# Scutellarin protects human retinal pigment epithelial cells against hydrogen peroxide (H_2_O_2_)-induced oxidative damage

**DOI:** 10.1186/s13578-019-0276-0

**Published:** 2019-01-21

**Authors:** Xin Hu, Xiaofang Wu, Bo Zhao, Yongyi Wang

**Affiliations:** Department of Ophthalmology, Huaihe Hospital, Henan University, No.8 of Baobei Road, Kaifeng, 475000 People’s Republic of China

**Keywords:** Proliferative vitreoretinopathy (PVR), Scutellarin, Retinal pigment epithelium (RPE) cells, Oxidative stress, JAK2/STAT3 signaling pathway

## Abstract

**Background:**

Proliferative vitreoretinopathy (PVR) is a severe blinding complication of retinal detachment surgery. Increasing evidence demonstrate that PVR is associated with oxidative stress. Scutellarin is a natural flavone compound that has been reported to have anti-oxidative activity. However, the effect of scutellarin on PVR remains unknown. In the current study, we assessed the effect of scutellarin on hydrogen peroxide (H_2_O_2_)-induced oxidative injury in human retinal pigment epithelium cells (ARPE-19).

**Methods:**

ARPE-19 cells were pretreated with different concentrations of scutellarin for 2 h, and then challenged with H_2_O_2_ (1 mM) for 24 h. The levels of reactive oxygen species (ROS), malondialdehyde (MDA) and superoxide dismutase (SOD) and glutathione (GSH) activity were measured to assess the level of oxidative stress. Flow cytometry was performed to detect the apoptosis rate of ARPE-19 cells. Expression levels of bcl-2, bax, cleaved-caspase-3, p-JAK2, JAK2, p-STAT3, and STAT3 were measured using western blot.

**Results:**

Our results revealed that scutellarin improved the cell viability of H_2_O_2_-induced ARPE-19 cells. Scutellarin alleviated the H_2_O_2_-induced oxidative stress in ARPE-19 cells, which was illustrated by reduced levels of ROS and MDA, accompanied by increased SOD activity and GSH level. The increased apoptosis rate of ARPE-19 cells caused by H_2_O_2_ induction was significantly decreased after scutellarin treatment. H_2_O_2_ treatment resulted in significant increase in bax expression and decrease in bcl-2 expression, while the changes in the expressions of bax and bcl-2 were reversed by scutellarin treatment. In addition, scutellarin promoted the activation of JAK2/STAT3 signaling pathway in H_2_O_2_-induced ARPE-19 cells. Suppression of JAK2/STAT3 signaling pathway abolished the protective effects of scutellarin on H_2_O_2_-induced ARPE-19 cells.

**Conclusion:**

These findings suggested that scutellarin was capable for alleviating H_2_O_2_-induced oxidative damage in ARPE-19 cells, which might be ascribed to the activation of JAK2/STAT3 signaling pathway.

**Electronic supplementary material:**

The online version of this article (10.1186/s13578-019-0276-0) contains supplementary material, which is available to authorized users.

## Introduction

Proliferative vitreoretinopathy (PVR) is a severe blinding complication of retinal detachment surgery that occurs in about 8–10% of clinical cases [1]. PVR is the major cause of treatment failure in individuals who undergo the surgery [1]. PVR is referred to as massive periretinal proliferation, which is characterized by the formation of epiretinal and/or sub-retinal membranes and traction of the reattached retina [2]. Although surgical techniques for PVR have been developed to reattach the detached retina, the visual outcome of the surgery is very poor [1]. Therefore, better understanding of the detailed molecular mechanisms underlying the pathogenesis of PVR may be helpful to explore new therapeutic strategy.

Oxidative stress refers to an imbalance between reactive oxygen species (ROS) production and antioxidant system, which may result in cell injury [3]. Increasing evidence has suggested that oxidative stress play a pivotal role in the pathogenesis of PVR [4]. The retinal pigment epithelium (RPE) cells are the main cell type involved in the progress of PVR. RPE cells have been found to be susceptible to oxidative damage since its unique phagocytotic function [5]. Increased oxidative stress may induce preferential damage to its mitochondrial DNA, leading to RPE cells death [5]. Therefore, inhibiting the oxidative stress in RPE cells is an effective approach for preventing the progress of PVR.

Scutellarin, a natural flavone compounds, has been reported to have multiple beneficial effects, such as anti-oxidative, anti-inflammation, anti-platelet, anti-coagulation, vascular relaxation, and myocardial protection properties [6]. New derivatives and formulations of scutellarin have been developed for the clinical use to treat stroke, myocardial infarction, and diabetic complications [6]. Additionally, scutellarin was reported to inhibit hypoxia-induced and high glucose-induced proliferation and vascular endothelial growth factor (VEGF) expression in human retinal endothelial cells (HRECs), suggesting that it might be a potential therapy for diabetic retinopathy [7]. Scutellarin exhibits an anti-angiogenic effect in high glucose-induced and hypoxia-mimetic agent-induced HRECs via inhibition of oxidative stress, enhancement of hypoxia-inducible factor (HIF)-1α, and reduction of VEGF secretion [7]. However, the effect of scutellarin on PVR has not been investigated. In the present study, we evaluated the effect of scutellarin on hydrogen peroxide (H_2_O_2_)-induced oxidative stress in human RPE cells.

## Materials and methods

### Cell culture and treatment

The human RPE cell line (ARPE-19) was obtained from ATCC (Manassas, VA). The ARPE-19 cells were maintained in Dulbecco’s modified Eagle’s medium (DMEM)/F12 (Invitrogen-Thermo Fisher Scientific, Waltham, MA, USA) supplemented with 10% heat inactivated fetal bovine serum (FBS, Invitrogen), 100 U/ml of penicillin (Sigma-Aldrich, St. Louis, MO, USA) and 100 µg/ml of streptomycin (Sigma-Aldrich). The cells were grown in a humidified incubator with 5% CO_2_ at 37 °C. For some experiments, cells were pretreated with different concentrations of scutellarin (25, 50, 100 μM) or AG90 (40 μM) for 2 h, and then challenged with H_2_O_2_ (1 mM) for 24 h.

### Small interference RNA (siRNA) treatment

Small interfere RNA targeting JAK2 (si-JAK2) and a control siRNA (si-NC) were provided by Biossci Company (Wuhan, China). ARPE-19 cells were transfected using Lipofectamine 2000 (Invitrogen, CA, USA) according to the manufacturer’s protocol.

### Cell viability assay

The ARPE-19 cells (5000 cells per well) were seeded in 96-well plates and incubated for 24 h. After different treatments, cell viability was measured using 3-(4,5-dimethylthiazol-2-yl)-2,5-diphenyltetrazolium bromide (MTT) method. In brief, 20 μl of the MTT reagent (Solarbio, Beijing, China) was added to each well and incubated for 4 h. After removing the cell medium, the formazan was dissolved by adding 200 μl dimethyl sulfoxide (DMSO). Finally, absorbance was measured at 570 nm using a microplate reader (Bio-Tek, Winooski, VT, USA).

### Measurement of intracellular reactive oxygen species (ROS) generation

The accumulation of intracellular ROS was examined using the ROS assay kit (Beyotime) according to the manufacturer’s instructions. Briefly, cells were grown in a 96-well plate and subjected to different treatments. Then the cells were incubated with 10 μM 2,7-dichlorofluorescein diacetate (DCFH-DA) at 37 °C for 20 min. The fluorescence intensity was measured using the fluorescence plate reader (BD Falcon, Bedford, MA, USA) at Ex./Em. = 488/525 nm.

### Detection of superoxide dismutase (SOD) activity, malondialdehyde (MDA) and glutathione (GSH) levels

After different treatments, SOD activity and the levels of MDA and GSH were measured using the commercial kits obtained from Sangon Biotech (Shanghai, China), according to the manufacturer’s instructions.

### Cell apoptosis assay

Flow cytometry was performed to quantify apoptosis rate using an Annexin V-FITC apoptosis detection kit (Bio-vision, Mountain View, CA, USA). The ARPE-19 cells with different treatments were collected and re-suspended in binding buffer at a density of 1 × 10^6^ cells/ml. FITC-AnnexinV (5 μl) and propidium iodide (PI, 5 μl) were gently mixed and added to the cells, followed by an incubation for 15 min at room temperature. The cells were analyzed by flow cytometry using a FACScalibur (Becton–Dickinson, Mountain View, CA, USA). Western blot analysis.

The total protein of ARPE-19 cells was extracted by using RIPA buffer (Solarbio) supplemented with protease and phosphatase inhibitors (Sigma-Aldrich, St. Louis, MO, USA). The protein concentration was measured by using BCA Protein Assay Kit (Beyotime Biotechnology, Shanghai, China). Aliquots of 20 μg protein were mixed with the loading buffer and the mixture was heated for 10 min at 95 °C. The protein samples were separated by 10–12% sodium dodecyl sulfate–polyacrylamide gel electrophoresis (SDS-PAGE). Afterward, the proteins were transferred electrophoretically to polyvinylidene difluoride (PVDF) membrane (Thermo Fisher Scientific). The membranes were then blocked with 5% skim milk in TBST buffer at room temperature for 1 h. Then, the membranes were incubated with antibodies against bcl-2, bax, cleaved-caspase-3, p-janus kinase 2 (JAK2), JAK2, signal transducer and activator of transcription protein 3 (STAT3), p-STAT3 overnight at 4 °C. Subsequently, the membranes were incubated with appropriate HRP-conjugated anti-rabbit IgG secondary antibody (Abcam, dilution of 1: 5000) for 1 h at 37 °C. Immunodetection was carried out using the ECL Plus detection system (Millipore, Billerica, MA, USA) according to the manufacturer’s instructions. Intensity of the bands was quantified using Image Lab™ Software (Bio-Rad Laboratories, Hercules, CA, USA).

### Statistical analysis

Results are expressed as mean ± standard error of the mean (SEM). The data were analyzed by student’s t-tests or one-way ANOVA using the SPSS 17.0 software (SPSS Inc., Chicago, USA). The comparisons were considered statistically significant when p < 0.05.

## Results

### Scutellarin improved cell viability in H_2_O_2_-induced ARPE-19 cells

**Fig. 1:**
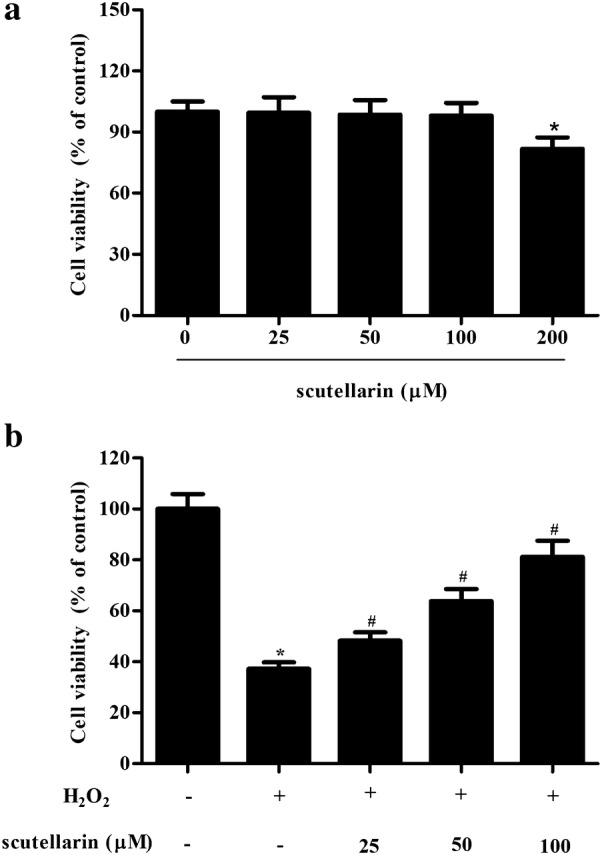
Scutellarin improved cell viability in H_2_O_2_-induced ARPE-19 cells. **a** Effect of scutellarin on cell viability of ARPE-19 cells. To evaluate the cytotoxicity of scutellarin on ARPE-19 cells, cells were treated with 0, 25, 50, 100, 200 μM scutellarin for 48 h. **b** Protective effect of scutellarin on H_2_O_2_-induced ARPE-19 cells. Cells were pretreated with different concentrations of scutellarin (25, 50, 100 μM) for 2 h, and then challenged with H_2_O_2_ (1 mM) for 24 h. Cell viability was detected using MTT assay. *p < 0.05 versus the control cells; ^#^p < 0.05 versus ARPE-19 cells exposed to H_2_O_2_

To evaluate the cytotoxicity of scutellarin on ARPE-19 cells, the cells were treated with a series of concentrations of scutellarin for 48 h. As shown in Fig. 1a, the results of MTT assay demonstrated that the concentration of 200 μM scutellarin produced a significant effect, while the concentration of 100 μM scutellarin did not affect the cell viability of ARPE-19 cells. Therefore, 25–100 μM of scutellarin was used in the following experiments. Next, we investigated the protective effect of scutellarin on H_2_O_2_-induced ARPE-19 cells. As shown in Fig. 1b, scutellarin pretreatment improved H_2_O_2_-caused reduction of ARPE-19 cells viability.

### Scutellarin inhibited H_2_O_2_-induced oxidative stress in ARPE-19 cells

**Fig. 2:**
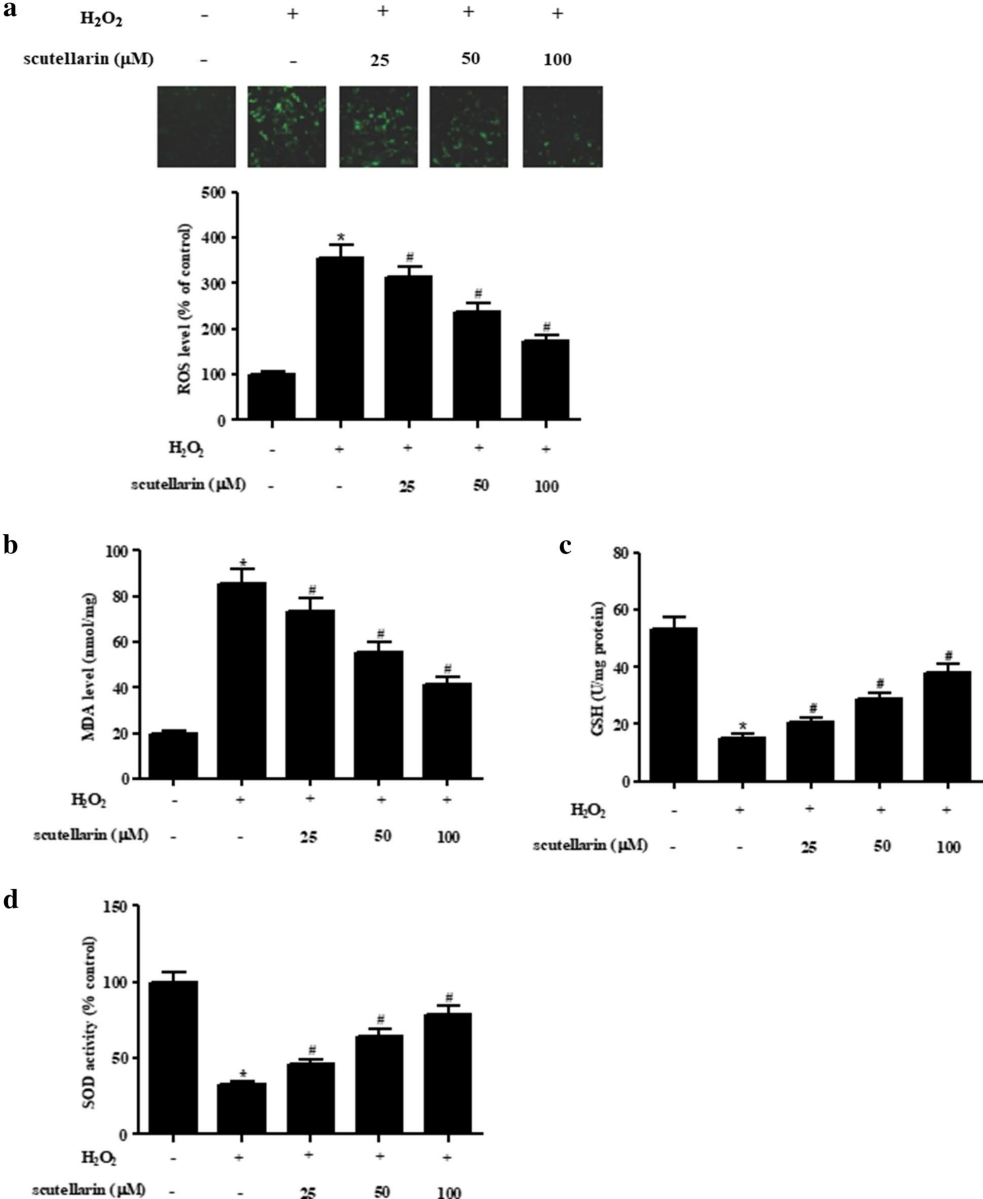
Scutellarin inhibited oxidative stress in H_2_O_2_-induced ARPE-19 cells. ARPE-19 cells were pretreated with different concentrations of scutellarin (25, 50, 100 μM) for 2 h, and then treated with H_2_O_2_ (1 mM) for 24 h. The levels of ROS (**a**), MDA (**b**) and SOD (**c**) and GSH activity (**d**) were measured to assess the level of oxidative stress. *p < 0.05 versus the control cells; ^#^p < 0.05 versus ARPE-19 cells exposed to H_2_O_2_

Then we assessed the effect of scutellarin on oxidative stress in H_2_O_2_-induced ARPE-19 cells. DCFH-DA was used to evaluate the intracellular ROS level, and the MDA level was detected using MDA ELISA kit. Figure 2a, b proved that ROS and MDA levels were markedly increased in ARPE-19 cells after H_2_O_2_ induction compared to the control cells. The increased ROS and MDA levels caused by H_2_O_2_ induction were reduced in the cells pretreated with scutellarin. Additionally, the SOD activity and GSH level were decreased in H_2_O_2_-induced ARPE-19 cells using SOD and GSH ELISA kits, when compared to control cells. However, the H_2_O_2_-caused changes in SOD activity and GSH level were attenuated by scutellarin (Fig. 2c, d).

### Scutellarin suppressed H_2_O_2_-induced apoptosis in ARPE-19 cells

**Fig. 3:**
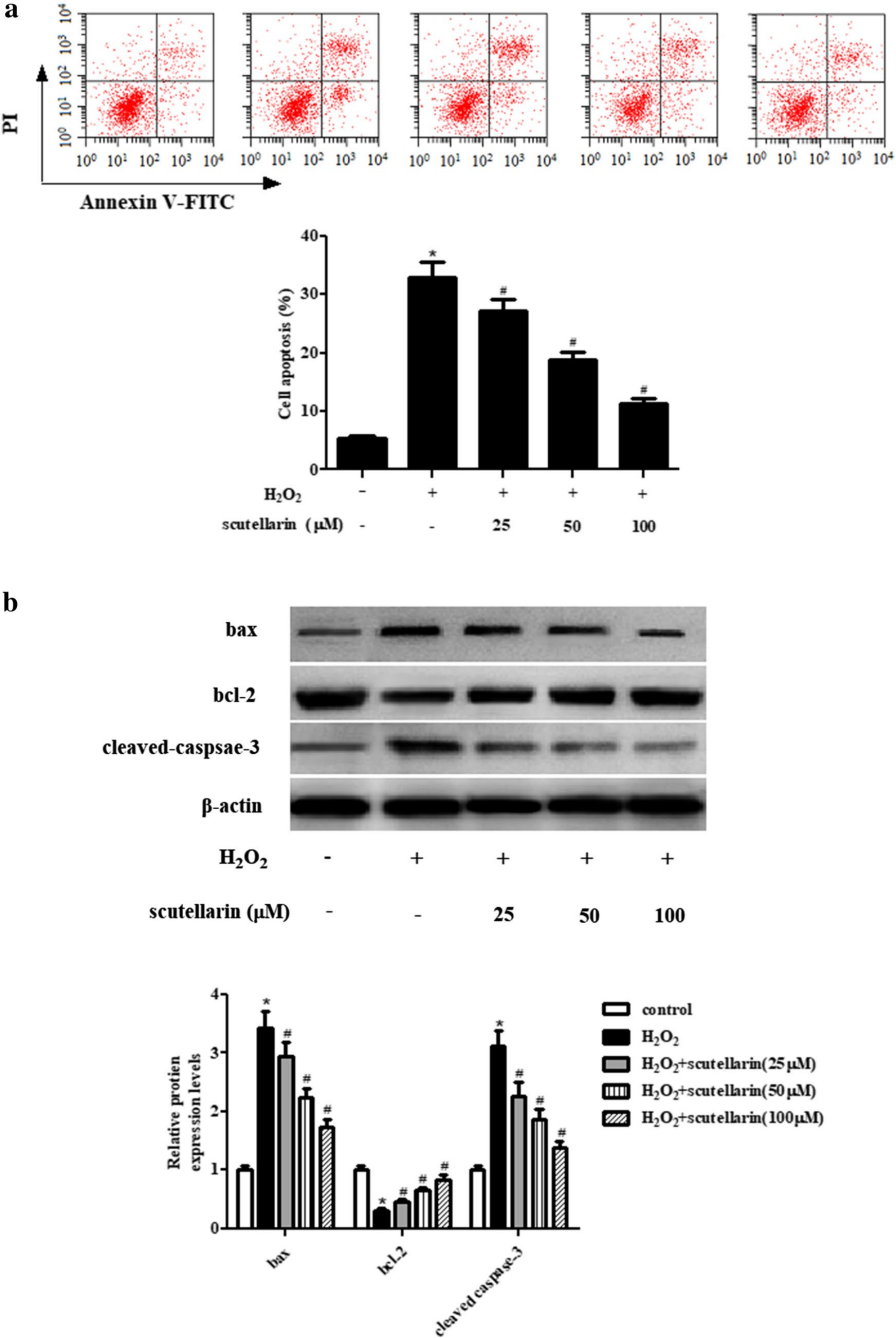
Scutellarin suppressed apoptosis in H_2_O_2_-induced ARPE-19 cells. ARPE-19 cells were pretreated with 25, 50, or 100 μM scutellarin for 2 h, followed by treatment with H_2_O_2_ (1 mM) for 24 h. **a** Flow cytometry was performed to detect the apoptosis rate of ARPE-19 cells. **b** Expression levels of bcl-2, bax, and cleaved-caspase-3 were determined using western blot. *p < 0.05 versus the control cells; ^#^p < 0.05 versus ARPE-19 cells exposed to H_2_O_2_

Next, flow cytometry was performed to detect the apoptosis rate of ARPE-19 cells with different treatments. As illustrated in Fig. 3a, the apoptosis rate was significantly increased in H_2_O_2_-induced ARPE-19 cells, however, the increase in apoptosis rate was suppressed by pretreatment with scutellarin. Besides, the expression levels of bcl-2, bax, and cleaved-caspase-3 were determined using western blot. The results in Fig. 3b indicated that H_2_O_2_ resulted in decrease in expression of bcl-2, and increase in expressions of bax and cleaved-caspase-3, while the alternations were mitigated by scutellarin pretreatment.

### Scutellarin elevated the activation of JAK2/STAT3 signaling pathway in ARPE-19 cells exposed to H_2_O_2_

**Fig. 4:**
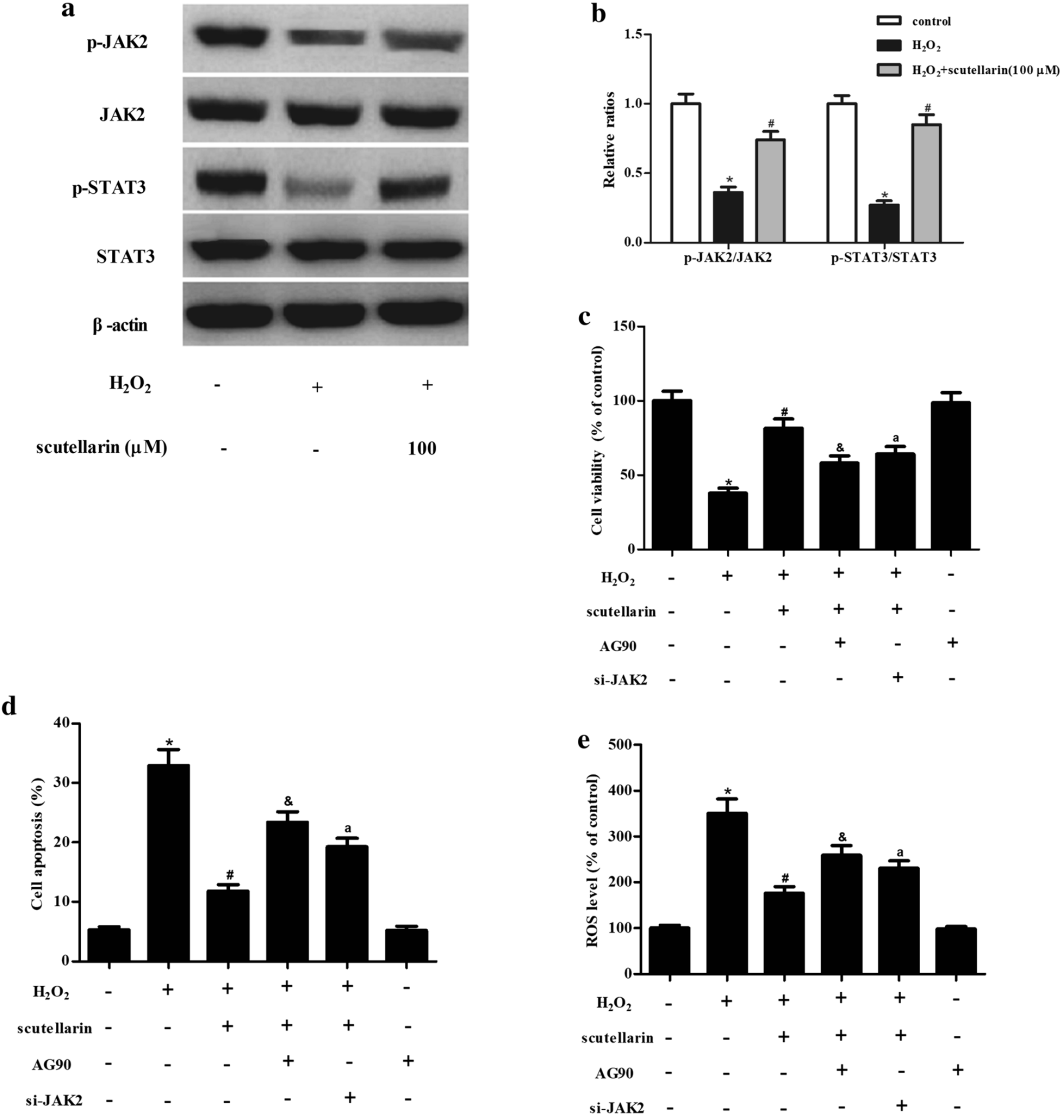
Scutellarin elevated the activation of JAK2/STAT3 signaling pathway in H_2_O_2_-induced ARPE-19 cells. **a** Effect of scutellarin on JAK2/STAT3 signaling pathway in H_2_O_2_-induced ARPE-19 cells. Cells were challenged with H_2_O_2_ (1 mM) for 24 h in the presence or absence of scutellarin (100 μM). Expressions of p-JAK2, JAK2, p-STAT3, and STAT3 were measured using western blot. **b** Quantification analysis of p-JAK2/JAK2 and p-STAT3/STAT3. Cells were pretreated with scutellarin (100 μM) and/or AG90 (40 μM) or si-JAK2 for 2 h, and then challenged with H_2_O_2_ (1 mM) for 24 h. **c**, **d** Effects of AG90 on cell viability and apoptosis. **e** Effect of AG90 on ROS level. *p < 0.05 versus the control cells; ^#^p < 0.05 versus ARPE-19 cells exposed to H_2_O_2_; ^&^p < 0.05 versus H_2_O_2_ + scutellarin (100 μM) group; ^a^p < 0.05 versus H_2_O_2_ + scutellarin (100 μM) group

It has been demonstrated that JAK2/STAT3 signaling pathway is involved in oxidative stress [8–10], therefore, we next evaluated the effect of scutellarin on this pathway. Western blot denoted that scutellarin elevated the H_2_O_2_-caused decrease in the expressions of p-JAK2 and p-STAT3, suggesting that scutellarin promoted the activation of JAK2/STAT3 signaling pathway in H_2_O_2_-induced ARPE-19 cells (Fig. 4a). We also found that scutellarin treatment only had no effect on p-JAK/JAK and p-STAT3/STAT expression levels (Additional file 1: Figure S1). Furthermore, treatment with the inhibitor of JAK2/STAT3 pathway, AG90, reversed the protective effect of scutellarin on cell viability of H_2_O_2_-induced ARPE-19 cells (Fig. 4c). Figure 4d showed that the inhibitory effect of scutellarin on cell apoptosis was mitigated by AG90. The decreased ROS level caused by scutellarin was reversed by AG90 (Fig. 4e). In addition, treatment with si-JAK2 also reversed the protective effects of scutellarin on ARPE-19 cells under H_2_O_2_ condition (Fig. 4c–e).

## Discussion

Recent investigations provide the new sight that PVR is considered as an uncontrollable plastic process caused by certain changes in the retina under oxidative stress [11]. Cicik et al. [4] determined the interleukin-8 (IL-8), nitric oxide (NO) and glutathione (GSH) profiles in vitreous humor and blood samples in patients with PVR. They found that the levels of IL-8, NO, and GSH in vitreous humor and plasma from patients with PVR are significantly higher than those of controls [4]. These results indicate that oxidative stress may be involved in the pathogenesis of PVR. Evidences from a variety of studies indicate that RPE cells are susceptible to oxidative damage [5]. Firstly, RPE is anatomically located between the sensory retina and choroid, allowing the RPE cells expose to a highly oxidative environment due to high oxygen partial pressure from choriocapillaris [5]. Secondly, RPE cells have a unique physiological function to phagocytose and digest photoreceptor outer segments, which are extremely rich in polyunsaturated fatty acids (PUFA). Oxidation of PUFA initiates a chain reaction producing an abundance of ROS, causing an additional oxidative burden [5]. Additionally, the process of RPE phagocytosis results in the generation of endogenous ROS [5]. Furthermore, RPE cells contain an abundance of photosensitizers, thereby, exposure to intense visible lights induces ROS generation [5]. Taken together, it is likely that RPE cells are more susceptible to oxidative damage.

Emerging evidence indicates that inhibition of oxidative stress in RPE cells may be helpful for alleviating PVR [12–14]. Ko et al. [12] reported that macrophage migration inhibitory factor (MIF) is involved in induction of the epithelial-mesenchymal transition (EMT) and related processes by oxidative stress in RPE cells, suggesting that it might contribute to the pathogenesis of PVR. Eupatilin, a pharmacologically active flavone, has been found to prevent H_2_O_2_-induced oxidative stress and apoptosis in human RPE cells, it thereby may be useful for the prevention or treatment of PVR [13]. Additionally, diosmetin protects against adriamycin (ADR)-induced retinal injury in vivo and in vitro via reduction of DNA damage and oxidative stress [14]. Scutellarin is a natural flavone that has been demonstrated to possess therapeutic effect against oxidative damage. Scutellarin protects ischemia–reperfusion (I/R) injury by reducing apoptosis and oxidative stress in cardiomyocytes [15]. Besides, scutellarin alleviates diosbulbin B (DB)-induced liver injury, which is attributed to inhibition of NF-κB-mediated hepatic inflammation and oxidative stress [16]. Scutellarin exhibits preventive and therapeutic effects in cerebral injury patients, and the effects are at least in part ascribed to the increased cellular antioxidant defense capacity [17]. In the present study, we evaluated the effects of scutellarin on H_2_O_2_-induced oxidative damage in ARPE-19 cells. The results showed that scutellarin improved cell viability, inhibited cell apoptosis and oxidative stress in H_2_O_2_-induced ARPE-19 cells.

JAK/STAT signaling pathway is a chain of interactions that is involved in multiple processes such as cell survival, proliferation, and death, as well as inflammation and oxidative stress [18, 19]. Recent evidence has demonstrated that JAK/STAT signaling pathway plays an essential role in H_2_O_2_-induced oxidative damage [20]. Yang and colleagues demonstrated that H_2_O_2_-induced oxidative injury of PC12 cells, while suppression of microRNA (miR)-146a relieves H_2_O_2_-induced cytotoxicity in PC12 cells via regulating MCL1/JAK/STAT pathway [21]. Huang et al. [8] demonstrated that H_2_O_2_ causes oxidative stress and apoptosis of myocardial cells through the reduction of JAK2/STAT3 signaling pathway. Secoisolariciresinol diglucoside exhibits anti-oxidative and anti-apoptotic effects through activation of the JAK2/STAT3 signaling pathway [8]. Scutellarin was found to inhibit the proliferation and induce the apoptosis of HepG2 cells via a STAT3 signal pathway [22]. A recent study reported that scutellarin modulates I/R injury-induced oxidative stress and apoptosis by enhancing JAK2/STAT3 pro-survival signaling [15]. These dual effects of scutellarin on cell apoptosis could attribute to different cellular contexts. In the current study, we further investigated whether the JAK2/STAT3 signaling pathway was implicated in the protective effects of scutellarin. Our results revealed that the reduction of JAK2/STAT3 signaling pathway in H_2_O_2_-induced ARPE-19 cells was elevated by scutellarin. Treatment with the inhibitor of JAK2/STAT3 signaling pathway attenuated the protective effects of scutellarin on H_2_O_2_-induced ARPE-19 cells.

## Conclusion

In conclusion, our study revealed that scutellarin protected ARPE-19 cells from H_2_O_2_-induced oxidative damage. Suppression of JAK2/STAT3 signaling pathway abolished the effects of scutellarin on cell viability, apoptosis and oxidative stress in H_2_O_2_-induced ARPE-19 cells. The protective effect of scutellarin was, at least in part, mediated by the activation of JAK2/STAT3 signaling pathway.

## Additional file

**Additional file 1: Figure S1.** Effect of scutellarin treatment only on JAK2/STAT3 signaling pathway in ARPE-19 cells. (A) ARPE-19 cells were treated with scutellarin (100 μM) for 24 h. Expressions of p-JAK2, JAK2, p-STAT3, and STAT3 were measured using western blot. (B) Quantification analysis of p-JAK2/JAK2 and p-STAT3/STAT3.

## Abbreviations

PVR: proliferative vitreoretinopathy
ROS: reactive oxygen species
MDA: malondialdehyde
SOD: superoxide dismutase
GSH: glutathione
RPE: retinal pigment epithelium
HRECs: human retinal endothelial cells
