# Supplementary figures and images for: Scutellarin protects human retinal pigment epithelial cells against hydrogen peroxide (H2O2)-induced oxidative damage
Source: Cell Biosci. 2019 Jan 21;9:12. doi: 10.1186/s13578-019-0276-0 (PMC6341765; doi:10.1186/s13578-019-0276-0)

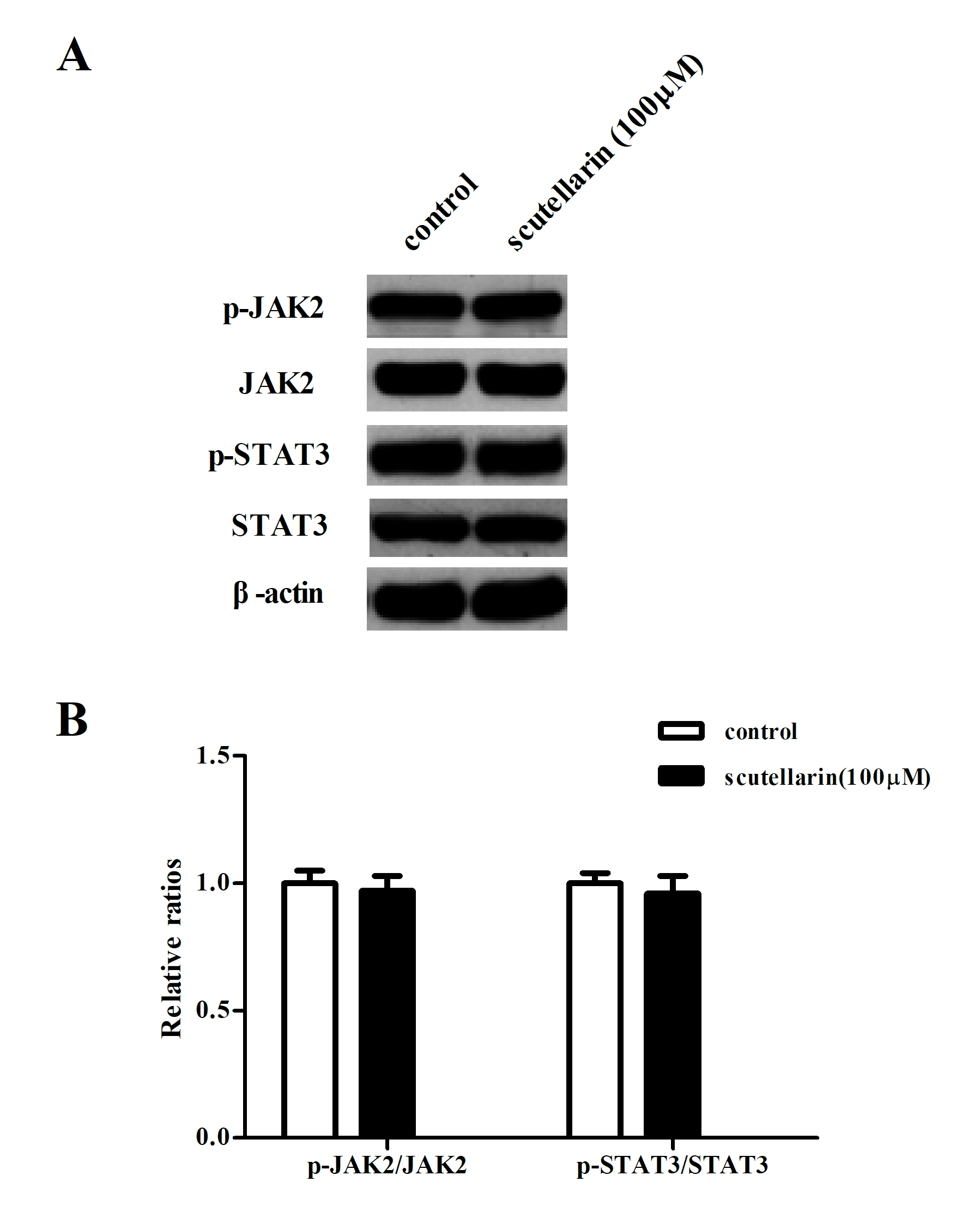

Supplement: Supplementary file 1 — Additional file 1: Figure S1. Effect of scutellarin treatment only on JAK2/STAT3 signaling pathway in ARPE-19 cells. (A) ARPE-19 cells were treated with scutellarin (100 μM) for 24 h. Expressions of p-JAK2, JAK2, p-STAT3, and STAT3 were measured using western blot. (B) Quantification analysis of p-JAK2/JAK2 and p-STAT3/STAT3. [file 13578_2019_276_MOESM1_ESM.tif]
